# Mitochondrial Dysfunction and DNA Damage in the Context of Pathogenesis of Atherosclerosis

**DOI:** 10.3390/biomedicines8060166

**Published:** 2020-06-18

**Authors:** Taisiia Shemiakova, Ekaterina Ivanova, Andrey V. Grechko, Elena V. Gerasimova, Igor A. Sobenin, Alexander N. Orekhov

**Affiliations:** 1 Institute of Translational Biomedicine, St. Petersburg State University, 199034 St. Petersburg, Russia; taisia070296@mail.ru; 2 Department of Basic Research, Institute for Atherosclerosis Research, 121609 Moscow, Russia; 3 Federal Scientific Clinical Center for Resuscitation and Rehabilitation, 109240 Moscow, Russia; noo@fnkcrr.ru; 4 Laboratory of Systemic Rheumatic Disorders, V.A. Nasonova Institute of Rheumatology, 115522 Moscow, Russia; gerasimovaev@list.ru; 5 Laboratory of Medical Genetics, Institute of Experimental Cardiology, National Medical Research Center of Cardiology, 121552 Moscow, Russia; igor.sobenin@gmail.com; 6 Laboratory of Angiopathology, Institute of General Pathology and Pathophysiology, 125315 Moscow, Russia; 7 Laboratory of Infection Pathology and Molecular Microecology, Institute of Human Morphology, 117418 Moscow, Russia

**Keywords:** atherosclerosis, mitochondrial dysfunction, mtDNA damage, oxidative stress, inflammation, antioxidants, endothelial cells

## Abstract

Atherosclerosis is a multifactorial disease of the cardiovascular system associated with aging, inflammation, and oxidative stress. An important role in the development of atherosclerosis play elevated plasma lipoproteins. A number of external factors (smoking, diabetes, infections) can also contribute to the development of the disease. For a long time, atherosclerosis remains asymptomatic, therefore, the search for early markers of the disease is critical for the timely management and better outcomes for patients. Mitochondrial dysfunction and mitochondrial DNA (mtDNA) damage appear to connect different aspects of atherosclerosis pathogenesis. To date, multiple lines of research have demonstrated the strong association of mitochondrial dysfunction with the development of various human diseases. Therapies aimed at restoring the mitochondrial function are being actively developed, and are expected to broaden the therapeutic possibilities for several chronic human diseases. The development of such therapies depends on our understanding of the functional roles of different mtDNA variants associated with one or another disorder, and the molecular mechanisms linking mitochondrial dysfunction with a given pathological feature. These questions are, however, challenging and require future intensive research. This review summarizes the recent studies and describes the central processes of the development of atherosclerosis, and shows their relationship with mitochondrial dysfunction. One of the promising therapeutic approaches for future atherosclerosis treatments is the use of mitochondria-targeted antioxidants. Future studies should focus on characterizing the mechanisms of mitochondrial involvement in cardiovascular pathologies to better direct the search for novel therapies.

## 1. Introduction 

Cardiovascular diseases (CVD) represent the most prevalent and socially significant group of diseases in the world. According to World Health Organization (WHO) statistics, in 2016, 17.9 million people died from CVD, which accounted for 31% of all deaths in the world [1]. The leading role in the development of CVD belongs to atherosclerosis, a chronic disease of the arteries of the elastic and muscular-elastic type. According to current knowledge, atherosclerosis is a consequence of an interplay of many factors leading to the fibrous plaque formation. A number of significant factors contributing to the development of the disease have been established, including smoking, hyperlipoproteinemia, arterial hypertension, diabetes, obesity, and viral and chlamydial infections accompanied by the development of a chronic inflammatory process in the arterial wall [2]. Atherosclerosis is closely associated with other chronic diseases, such as coronary heart disease, cerebral ischemia, and stroke. Currently, general belief places atherosclerosis among age-associated disorders. However, the development of noninvasive imaging methods and increasing availability of diagnostic facilities made it possible to demonstrate that the disease manifests itself already in the second decade of life. Asymptomatic lesions have been described in young adults [3]. Moreover, 75% of men and 38% of women suffer from this disease after 30–35 years [2].

Pathological processes that develop in the vascular wall in atherosclerosis proceed sequentially for a long period of time. The artery remains functional until the atherosclerotic plaque obstruction reaches about 40% of the vessel lumen, due to the stretching of the outer elastic membrane [4]. Nevertheless, already at the preclinical stage of the disease, certain arterial pools can be affected. Therefore, one of the priorities of clinical cardiology is the early diagnosis of the disease. This would not only prevent the development of complications, but also save patients’ lives and their ability to work. According to the current understanding, one of the earliest events in atherosclerosis is vascular endothelial dysfunction [5]. Local disturbance of the endothelium is accompanied by its activation and initiation of the inflammatory response with recruitment of circulating immune cells to the future lesion site. The disturbed endothelium also becomes more permeable thus facilitating the entry of lipoprotein particles into the arterial wall. The triggers of the endothelial dysfunction are not yet fully understood. They can include both the external stimuli, such as disturbances of the laminar blood flow caused by the vessel bends and branching, and the presence of dysfunctional cells within the endothelial layer itself. Better understanding of these factors is crucial for finding effective preventive strategies against atherosclerosis.

In the last century, numerous hypotheses have been proposed to explain the origins and progression of the atherosclerotic process. Among them are such popular concepts as infection theory, thrombolipid theory, cholesterol theory, endothelial damage theory, peroxide, and infiltration theory. According to these theories, atherogenesis is based on the following phenomena: viral and bacterial infection; the formation of a blood clot in the vessel wall; violation of lipid metabolism, leading to the formation of atherosclerotic plaques; damage to the endothelium, to which it responds with inflammation; free radical oxidation; and “primary lipoid infiltration of the inner lining of the arteries—lipoidosis—with subsequent development of connective tissue (sclerosis)” [6,7,8,9,10]. However, to date, not one of them, even the most popular and reasoned, is universally recognized and conclusively proven. In this review, we will present the hypothesis of atherosclerosis development due to mitochondrial dysfunction in the arterial wall cells and propose possible mechanisms of pathology development.

## 2. Atherosclerosis as an Inflammatory Process

There is currently little doubt that atherosclerosis is tightly linked to sterile inflammation to such extent that it can be classified as a chronic inflammatory disease. Under normal conditions, inflammation mediates only a temporary incapacitation of tissues, followed by tissue restoration and remodeling. however, under pathological conditions, the process becomes chronic and ends with prolonged dysfunction [11]. Uncovering the factors leading to inflammation chronification is an important step towards better understanding the pathogenesis of different chronic diseases.

Signs of a locally visible inflammatory process in atherosclerosis can be present from the earliest stages of lesion development in the affected vessel wall. The immune process involves immunocompetent cells: T- and B-lymphocytes (the main components of the adaptive immune response), possibly mast cells and other cell types. But the key role belongs to monocytes and macrophages that contribute significantly to lipid accumulation and atherosclerotic plaque growth. Adhesion of circulating monocytes to activated endothelial cells is considered to be the earliest stage of inflammation [12]. Vascular damage is accompanied by the expression of adhesion molecules such as intracellular adhesion molecule-1 (ICAM-1), vascular cell adhesion molecule-1 (VCAM-1) and platelet/endothelial cell adhesion molecule-1 cells (PECAM-1), integrins and L, E, P selectins. The association of endothelial adhesion molecules with blood monocytes and lymphocytes ensures differentiation and migration of these cells into the subendothelial space of blood vessels [13]. Continuing immune response is accompanied by the release of pro-inflammatory cytokines and chemokines that further aggravate it by attracting more immune cells to the lesion site. It is known that disruption of the course of the innate immune response can influence the severity of atherogenesis [14].

The next stage of the inflammatory process in atherosclerosis is differentiation of monocytes into macrophages. Part of the monocytes entering the vascular wall under the influence of macrophage colony-stimulating factor (M-CSF), granulocyte-macrophage colony-stimulating factor (GM-CSF) and other factors secreted by the endothelium become differentiated. Differentiated macrophages can start internalization of large quantities of lipids, therefore becoming foam cells or focus more on maintaining the inflammatory response. Macrophages express scavenger receptors and participate in active phagocytosis and release multiple pro-inflammatory factors into the plaque milieu [15]. The spectrum of signaling molecules produced by activated macrophages is broad and includes interleukin (IL)-1, 2, 6, 7, 8, 18, tumor necrosis factor-α (TNF-α), interferon-α (IFN-α), monocyte chemotactic protein-1 (MCP-1), MCP-4, CD40 ligand (CD40L), parathyroid hormone-related protein (PTHrP), osteopontin, cyclooxygenase-2 (COX-2) and matrix metalloproteinases. The main mechanism of cellular response to the release of pro-inflammatory cytokines is the nuclear factor-κB (NF-κB) pathway, which regulates the expression of pro-inflammatory cytokine genes, adhesion molecules, growth factors, and inducible enzymes (cyclooxygenase and nitric oxide synthase), and also affects the level of reactive oxygen species (ROS) in the cell [16].

A considerable part of macrophages captures the excess of modified lipoproteins and turns into foam cells. It is likely that these cells engulf lipoprotein particles through unspecific phagocytosis as opposed to receptor-mediated endocytosis. Phagocytized lipoproteins escape the normal degradation route in the lysosomes and accumulate as undegraded lipid inclusions (droplets) in the cytoplasm hence becoming foam cells. Such cells contribute to the plaque growth containing the intracellular lipid pool of the plaque. It should be noted, however, that macrophages are not the only cell type present in the arterial wall that are capable of such accumulation [17]. Mast cells that penetrate the arterial wall secrete multiple pro-inflammatory cytokines that promote proliferation of smooth muscle cells and regulate the production of the extracellular matrix, but also activate metalloproteinases that cause degradation of the latter [18].

According to current understanding, local and systemic inflammation mediates all stages of the formation of fibrous plaque, its development and degradation. The transition from local acute inflammation to chronic inflammation (the process of chronification) is likely to play a key role in the development of atherosclerotic plaque [19]. Such transition can be dependent on different processes, but mitochondrial dysfunction is likely to be one of the most potent of them. As explained further, dysfunctional mitochondria act as sources of oxidative stress and as pro-inflammatory agents through the release of harmful molecules, damage-associated patterns (DAMPs) and cell and tissue damage.

## 3. Oxidative Stress and Atherosclerosis

Oxidative stress plays a crucial role in the development of endothelial dysfunction and is a potent modulator of the inflammatory response, cell growth and differentiation, apoptosis and changes in vascular tone [20]. It is therefore relevant for several important processes leading to atherosclerotic plaque development. The lack of success in using systemic antioxidant agents for prevention and treatment of atherosclerosis highlights the importance of better understanding of oxidative stress mechanisms in the disease context [21].

The main enzymes that generate ROS in the vascular wall cells are nicotinamide adenine dinucleotide phosphate (NADPH) oxidases, xanthine oxidase, mitochondrial enzymes, such as respiratory chain complexes, lipoxygenase, and myeloperoxidase. In the vascular wall, ROS induce proliferation of smooth muscle cells, apoptosis of endothelial cells, and increase the activity of matrix metalloproteinases, therefore providing input to plaque destabilization [22]. Generally, ROS production increases in response to pro-inflammatory cytokines. Some cytokines, for example, TNF-α, are able to increase the expression of NADPH oxidases. Moreover, under conditions of oxidative stress, nitric oxide (NO) synthase acquires the ability to synthesize an oxygen ion that reacts with nitric oxide to form peroxynitrite, which has oxidizing and nitrating properties. Thus, the anti-atherogenic activity of endothelial NO synthase is inhibited [23].

Peroxidase enzymes, such as xanthine oxidase, play the trigger role in the formation of superoxide radicals that can give rise to other ROS. Xanthine oxidase is activated in tissues with significant narrowing of vessels during stress, especially with damaged microvessels. Excessive ROS generation causes damage to microvessels increasing their permeability and also affects the organ parenchyma. Superoxide anion and myeloperoxidase are also key compounds that take part in atherosclerosis pathogenesis and associated inflammation. Increased myeloperoxidase activity correlates with the severity of atherosclerosis and the level of calcium in the walls of the coronary arteries [24].

Elevated blood fibrinogen level is a diagnostic and prognostic marker of atherosclerosis and other inflammatory diseases. Oxidized fibrinogen modifications are associated with impaired hemostatic system connected with endothelial dysfunction, which leads to impaired platelet and erythrocyte aggregation, and to increased secretion of cytokines [25].

Mechanisms of free radical utilization include nonenzymatic reactions involving low molecular weight “radical traps” and enzymatic reactions. Key antioxidant enzymes include superoxide dismutase and glutathione peroxidase. Enzymes utilize ROS or prevent their formation, and also stop the labile products of free radical oxidation, which can contribute to formation of secondary organic radicals. The primary products of free radical oxidation—lipoperoxides, have been discovered in atherosclerotic plaques in the early studies. The lack of equilibrium between the oxidative and antioxidant systems together with hyperlipidemia creates favorable conditions for the initiation of lipid peroxidation processes [26].

## 4. Mitochondrial Reactive Oxygen Species

Mitochondria are both the main target of ROS and their key source. Mitochondrial dysfunction can lead to increased production of ROS, which can mediate malfunctioning of mitochondrial DNA (mtDNA), the accumulation of oxidized low-density lipoprotein (LDL) in the vessel wall and stimulation of atherogenesis. The danger of ROS lies in the fact that they trigger a vicious circle: ROS damage mitochondria, while damaged mitochondria produce more ROS. The ability of mitochondrial ROS to induce an inflammatory response connects the oxidative and inflammatory theories of atherogenesis [27].

Mitochondrial ROS are of particular interest because of their importance for the transmission of cellular signals, changes in cell function, and the role in vascular diseases. Complexes I and III of the respiratory chain of the mitochondria are well characterized as ROS sources. Moreover, other sources have also been identified, such as nicotinamyl adenine dinucleotide phosphate oxidase 4 (NOX4), the monoamoxidase enzyme family, and the p66Shc protein growth factor adapter. NOX4 is widely represented in the mitochondria of many tissues, including the endothelium, where it plays an important role in ROS signaling processes, angiogenesis, and adaptive responses to hypoxia, but also promotes aging of the endothelial cells, inflammation and cell stress [28]. The monoamine oxidase family of enzymes are localized on the outer mitochondrial membrane and generates ROS during the catabolism of catecholamines. The p66Shc protein growth factor adapter functions in mitochondrial signaling. p66Shc generates hydrogen peroxide by oxidation of cytochrome c. Another mitochondrial ROS regulator is the mitochondrial ATP-sensitive potassium channel [27].

A significant contribution to the management of excess mitochondrial ROS is made by the altered potential of the mitochondrial membrane. Membrane depolarization can activate ROS production by increasing the activity of complexes I and III. Hyperpolarization of the membrane also leads to hyperproduction of ROS, which is especially important in conditions of surplus nutrients and low energy requirements [29].

Mitochondrial ROS enhance the oxidation of LDL and proteins, can cause significant nuclear and mtDNA damage and death of smooth muscle and endothelial cells. They also act as signal transduction molecules that trigger the release of inflammatory cytokines. The effects of increased mitochondrial ROS involve endothelial dysfunction, vascular inflammation, and the accumulation of oxidized LDL in the arterial wall. All these processes are atherogenic and lead to aggravation of the plaque phenotype [30]. Thus, mitochondrial dysfunction may turn out to be a key link in the pathogenesis of atherosclerosis, and elevated ROS is a likely mediator of this process.

## 5. Low Density Lipoproteins in the Pathogenesis of Atherosclerosis

Disbalance of plasma lipoproteins is one of the key elements of the pathogenesis of atherosclerosis. Biochemical changes in the lipoprotein spectrum for a long time may remain the only manifestation of the disease. The mortality from atherosclerosis-related conditions and the severity of complications were shown to be dependent on the level of total cholesterol and LDL-cholesterol in the blood plasma [31]. Population studies have demonstrated a direct relationship between the risk of developing atherosclerosis and an increased level of LDL-cholesterol and apolipoprotein B100 (apoB) (the main structural component of LDL). Penetration and subsidence of apoB in the arterial wall is a trigger event in the initiation of the inflammatory process and atherogenesis [32]. However, despite the strong association of cholesterol and lipoprotein level with atherosclerosis, many people with high cholesterol do not develop the disease [33]. Numerous studies have demonstrated that the main danger in the pathogenesis of atherosclerosis is represented by modified lipoproteins [34]. Several subfractions of LDL can be distinguished using physical–chemical methods [35]. Within the LDL fraction, small dense LDL (sdLDL), electronegative LDL (LDL(−)) and chemically modified LDL appear to be the most relevant for atherogenesis. It is likely that the atherogenic sdLDL and LDL(−) belong to the same fraction of LDL that was chemically modified in the blood (desialylated) [36,37]. Small LDL particles are known to cross the endothelial barrier 1.7 times faster than large ones and to bind more easily to proteoglycans increasing the residence time in the arterial wall [15]. Further chemical reactions, such as oxidation, are facilitated in such particles. Lipid peroxidation leads to the formation of oxidized LDL (oxLDL), for which the lack of balance between the oxidative and antioxidant systems creates favorable conditions. The oxidative modification of LDL is a reorganization of the phospholipid shell with exposure of phosphorylcholine and adduction of aldehydes (malonic aldehyde) to apoB [38].

LDL oxidation is likely to occur in the subendothelial space of the arterial wall in the presence of transition valence metals (iron and copper) and the oxidation products of macrophages, T cells, and foam cells. Monocytes differentiated into macrophages in the subendothelial layer express receptors that bind oxLDL, due to which the capture of oxLDL is facilitated as compared to that of native LDL [39]. A macrophage scavenger receptor CD36 can bind oxLDL and mediate the negative regulation of angiogenesis, differentiation of monocytes, accumulation of lipid in macrophages, fatty acid transport, and apoptosis of cells, which contributes to inflammation [40]. Another scavenger receptor, SR-A, regulates receptors internalization, cell adhesion and apoptosis [41]. SR-BI involves in processes of lipid uptake and cholesterol regulation [42]. Foam cells that subsequently undergo apoptosis release cholesterol crystals that further provoke inflammation due to their cytotoxicity [38].

Formation of anti-LDL autoantibodies can be considered as an important step in atherosclerosis pathogenesis [43]. Antibodies to oxLDL can be a marker of lipoprotein oxidation at the tissue level in the vascular wall. During oxidation of LDL, apoB is also oxidized, which favors the connection of the formed aldehydes with the amino group of apoB, inducing the expression of specific immunological epitopes on the surface of the protein. It was shown that, by interacting with LOX-1, oxLDL induces functional expression of tissue factor in T lymphocytes in vitro [44]. It is believed that oxidized cholesterol phospholipids and esters are recognized by TLR-associated mechanisms of innate immunity as markers of damage. Antibodies to oxLDL may be independent predictors of atherogenesis [38].

It has been suggested that oxidized lipids and lipoproteins belong to the class of endogenous DAMPs and have common structural motifs with microbial pathogen-associated molecular patterns (PAMPs). Therefore, oxidized lipids can activate the same pattern recognition receptors (PRR) on immune and vascular cells. It is believed that a reaction in the form of an immune response to these oxidized lipids can turn out to be both physiological and pathophysiological under different conditions [45,46].

Thus, oxidative stress is a critical factor underlying vascular aging and atherogenesis. It is closely associated with mitochondrial dysfunction and inflammation. The contribution of these processes to the pathogenesis of atherosclerosis is widely recognized, but the cause–effect relationships between the phenomena are not fully understood.

## 6. Mitochondrial Dysfunction in Atherosclerosis

As discussed previously, mitochondrial dysfunction mediates various chronic diseases, including atherosclerosis [47]. It is known that disruption of mitochondrial function contributes to increased ROS generation, damage of mtDNA, decreased mitochondrial respiration, and lipodystrophy, a condition in which the oxidation of fatty acids is impaired, adipogenesis decreases, adipocyte apoptosis increases, which leads to a general decrease in adipocyte reserves [48].

**Figure 1:**
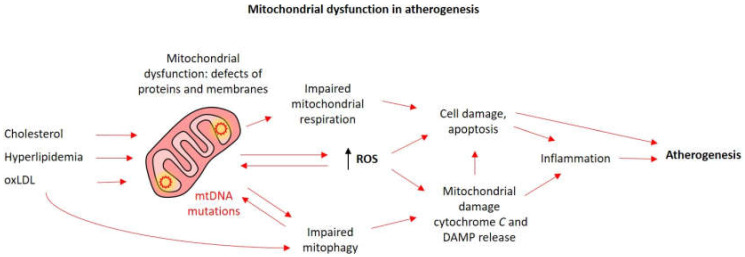
Mitochondrial dysfunction in atherosclerosis. External damaging stimuli (arrows), such as excessive lipids and oxLDL and internal accumulation of mtDNA mutations lead to progressive mitochondrial dysfunction. Impaired mitophagy hinders mitochondrial turnover and replenishment of functional organelles. Increased reactive oxygen species (ROS) formation further contributes to mtDNA mutagenesis and mitochondrial damage. These processes eventually lead to mitochondrial damage with the release of damage-associated molecular patterns (DAMP) and apoptosis-triggering molecules, causing cell death and inflammation and contributing to atherogenesis.

Excessive ROS make a huge contribution to mtDNA damage, but this mechanism may not be the only one linking mitochondrial abnormalities with atherosclerosis. It was shown that mtDNA defects weaken the expression of respiratory complexes and mitochondrial respiration in smooth muscle cells, monocytes, and macrophages. ATP reduction is associated with apoptosis and inhibition of cell proliferation. The result of these processes is pro-atherogenic phenomena: hyperlipidemia, apoptosis of smooth muscle cells and monocytes, isolation of inflammatory cytokines, and plaque vulnerability (Figure 1) [48].

Mitochondrial defects associated with atherosclerosis also include violations of mitophagy, a type of autophagy, a mechanism aimed to eliminate damaged cell components, which helps to maintain intracellular homeostasis [49]. Studies of atherosclerotic plaque cells for autophagy markers p62 and LC3-II isolated from human samples and observed in mouse models showed a decrease in autophagy or its dysfunction during the development of atherosclerosis. The degree of plaque destabilization correlated with the amount of LC3-II [50,51].

**Figure 2:**
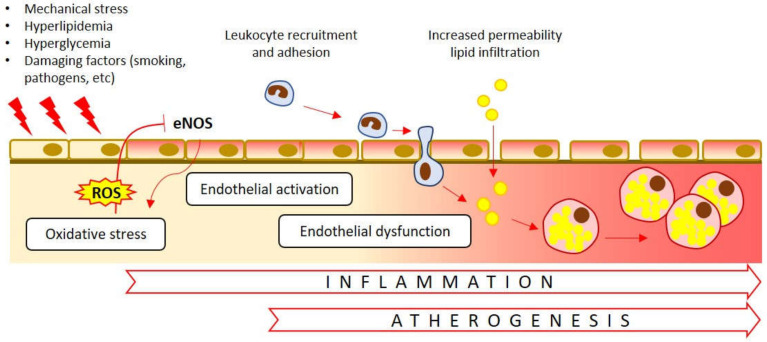
Endothelial dysfunction in atherosclerosis. Oxidative stress causes inhibition (T-bar) of the endothelial nitric oxide synthase (eNOS). Dysfunctional eNOS, in turn, further contributes to ROS formation (arrows). A number of other internal and external damaging factors impact the endothelial cells. Activation of the endothelial cells is associated with recruitment and adhesion of the immune cells, their penetration into the subendothelial space and initiation of the inflammatory response. Increased permeability of dysfunctional endothelium facilitates lipid entry into the arterial wall.

Mitochondria are dynamic organelles that undergo cycles of fission and fusion. In this process, defective organelles or parts of the organelles are isolated and destined for degradation through mitophagy, while functional parts can be joined together to form functional mitochondria [52]. Mitophagy is one of the methods of cell survival in which the destruction of defective mitochondria occurs, that prevents their accumulation, leading to cell degeneration. Mitochondrial turnover and mitophagy are critical for the normal functioning of the cardiovascular system, which is characterized by high energy consumption. In the context of atherosclerosis, mitophagy helps to reduce inflammation in macrophages, reduce degradation of oxLDL in the endothelial cells, and limits apoptosis of smooth muscle cells [53,54]. Activation of mitophagy by oxLDL and the administration of melatonin counteracts the progression of atherosclerosis by stabilizing atherosclerotic plaque [55]. In contrast, defective mitophagy can stimulate pro-inflammatory responses mediated by mitochondrial ROS-induced nod-like receptor-3 (NLRP3) signaling and contribute to lipotoxicity [56]. Disruption of mitophagy in cells can be caused by various pro-atherogenic stimuli: 7-ketocholesterol, oxidized lipids, 4-hydroxynonenal, osteopontin and TNF-α, platelet-derived growth factor (PDGF) (Figure 2) [54].

The content of mitochondria in the cell depends on the balance between mitochondrial biogenesis and mitophagy. The main regulator of mitochondrial biogenesis is peroxisome proliferator-activated receptor gamma coactivator 1-alpha (PGC-1α). It is expressed under the control of many factors: NO, sympathetic beta-receptor activation, calcineurin, cAMP, AMP-activated protein kinase, p53 and calcium/calmodulin-dependent protein kinase. In general, PGC-1α is activated in conditions of increased energy demand and serves to increase the ability of cells to produce ATP. PGC-1α-induced mitochondrial biogenesis is believed to protect against oxidative stress by supplying intact mitochondria that produce less ROS [27].

Certain mtDNA polymorphisms were found to be possible mediators of predisposition to atherosclerosis through mitochondrial dysfunction. Thus, polymorphisms of electron transport chain-related genes can lead to variations in the intensity of ATP synthesis, ROS formation activity, phosphorylation, and oxidation by-products. It has long been known that mtDNA is more sensitive to damage due to absence of protective histone packaging and relatively primitive repair system [57]. mtDNA oxidative damage leads to mitochondrial dysfunction and further formation of oxidizing agents. Such a “vicious circle” model was studied in mice knocked out with the adenine nucleotide translocator 1 (ANT1), in which increased oxidative stress correlated with mtDNA damage [58]. However, excessive ROS formation is not the only source of mtDNA damage. In mice knocked-out by gamma polymerase, mtDNA mutations were formed at the background of low oxidative stress, as evidenced by the activity of antioxidant systems. Without a clear increase in the ROS level, the effects of mtDNA damage were manifested: mice knocked-out by apoE and gamma polymerase demonstrated apoptosis of smooth muscle cells and monocytes, and decreased cell proliferation [48].

Thanks to mitochondrial turnover and compensatory mechanisms, mitochondrial damage for a long time may not be apparent at the functional level. Therefore, the search for markers of early mitochondrial disorders during the preclinical stage of diseases associated with mitochondrial dysfunction is an important line of research.

## 7. mtDNA Mutations

Human mtDNA encodes 37 genes, including 2 rRNA, 22 tRNA, and 13 proteins. Proteins encoded by mitochondria, including subunits of the respiratory complexes are indispensable for oxidative phosphorylation. rRNA and tRNA obtained from mtDNA are also necessary for the synthesis of mitochondrial proteins [59]. Damage to mtDNA reduces the efficiency of oxidative phosphorylation and ATP production. Mutation of mitochondrial genes leads to tRNA dysfunction and impaired respiratory protein synthesis. These processes decrease the efficiency of oxidative phosphorylation of ATP production [60].

Defects of mtDNA have been shown to play a crucial role in the pathogenesis of all stages of atherosclerosis development and can be considered as a preclinical marker of the disease [61]. In atherosclerosis, damaged mtDNA is observed both in the walls of blood vessels and in circulating immune cells. Defects of mtDNA are known to lead to pro-atherogenic processes: inflammation, apoptosis, and aging of cells. And the early appearance of defective mtDNA in the pathogenesis of atherosclerosis indicates the causative role of this process [48]. The main contribution to changes in mtDNA is likely to be made by oxidative stress, to which mtDNA is more susceptible than nuclear DNA. Mitochondrial oxidative stress promotes mutations (insertions, point mutations, deletions), changes in the number of mtDNA copies and haplogroups of mtDNA. This leads to increased production of ROS, decreased ATP level, and mitochondrial damage. mtDNA mutations can be caused by mtDNA damage or spontaneous errors in DNA replication [62].

The exact mechanism of the participation of mtDNA variants in damage processes remains unknown. However, the list of mtDNA variants associated with the disease or more severe disease phenotype is currently growing, allowing to expect uncovering of part of such mechanisms by future studies. For instance, leukocyte mtDNA mutations that cause the development of atherogenesis have been described (Table 1) [63]. MtDNA mutations can lead to a decrease in the synthesis of enzymes that generate energy and tRNA, leading to oxidative stress. All these are risk factors for the development of the atherosclerotic process. In age-associated atherosclerosis, oxidative stress and accumulating mtDNA damage play an important role, although the contribution that aging makes to the process is not yet fully understood [62].

In 2017, the Iranian population of patients with atherosclerosis was examined for the presence of mitochondrial mutations (on samples of peripheral blood). One new heteroplasmic mutation m.5725T>G in stem of tRNA gene and three previously registered for other diseases were identified: m.5568A>G in tRNA in T-loop, m.5711A>G D-loop of tRNA gene and m.12308A>G tRNA-Leu (CUN) gene. Detected mutations can change the structure of tRNA and affect the functioning of mitochondria [64].

In 2020, a number of mitochondrial mutations were described (isolated) based on an analysis of 29 articles. As a result, an association was found between mtDNA mutations and the development of atherosclerosis. The following mtDNA mutations are m.617G>A (tRNA-Phe), m.3243A>G (tRNA-Leu), m.8794T>C (ATP6), m.8839G>C (ATP6), m.3316G>A (subunit 1 of respiratory chain complex-I), have been associated with various manifestations of atherosclerosis, such as carotid artery stenosis, ischemia, vascular dementia, coronary artery disease, posterior cerebral artery stenosis, renal arteriolosclerosis, coronary sclerosis, and carotid artery occlusion [65].

Changes of copy numbers of mtDNA is also relevant for atherosclerosis, and the number of mtDNA copies per organelle can reflect the mitochondrial functionality. Higher copy numbers of mtDNA were shown to be associated with healthier aging [66].

It is known that methylation of mtDNA is associated with aging of the endothelial cells. CVD patients show more methylated mtDNA of genes encoding cytochrome-c oxidase and genes involved in ATP synthesis [67]. The level of mtDNA methylation can be a valuable, noninvasive CVD biomarker, which is also relevant for atherosclerosis.

Thus, mtDNA mutations, an altered number of copies, mtDNA haplogroup and epigenetic changes, all these can contribute to mitochondrial damage and lead to atherosclerosis.

## 8. Endothelial Dysfunction in Atherosclerosis

Endothelial dysfunction is one of the first signs of atherogenesis, which is accompanied by a decrease in the secretion of NO, one of the main regulators of the vascular tone, which limits the synthesis of adhesion molecules and chemokines and prevents platelet aggregation. Endothelial NO is an anti-inflammatory and antithrombogenic factor [30].

Oxidative stress associated with mitochondrial dysfunction and Nox-dependent formation of ROS plays a central role in triggering the development of endothelial dysfunction (Figure 2). ROS can interact with NO, forming peroxynitrite, which azoates the proteins, thus provoking mtDNA damage and destruction of mitochondrial integrity. Peroxynitrite dose-dependently inhibits mitochondrial protein synthesis, which leads to a decrease in the ATP of the cell and in general the number of mitochondria [30]. Oxidative stress is able to inactivate the endothelial NO synthase (eNOS) therefore reducing the amount of generated NO. In addition, dysfunctional (fragmented) NOS promotes ROS production [68,69].

Endothelial cell death is an important factor in the development of atherosclerosis. During the apoptosis of the endothelial cells, the redistribution of phosphatidylserine on the cell surface and the loss of anticoagulant surface components (thrombomodulin, heparan sulfate and tissue pathway inhibitor) increases the procoagulant properties of the endothelium. The involvement of endothelial cell apoptosis in the progression of atherogenesis is supported by the fact that the course of the disease is facilitated by statin therapy. Statins inhibit isoprenoid synthesis, in addition to lowering total cholesterol and LDL cholesterol, which affects inflammation, proliferation, migration, and cell survival [70]. During atherogenesis, cell death processes are induced by several mechanisms: oxLDL promote apoptosis of endothelial cells through secretion of ROS or in a Fas ligand-dependent manner [71]. Hyperglycemia induces apoptosis by PI3K/AKT signaling, which triggers NF-κB-dependent regulation of COX-2 and activation of caspases [72]. Mitochondrial calcium overload, loss of mitochondrial membrane potential, and release of cytochrome c are involved in apoptosis induced by oxLDL cholesterol [56].

## 9. Prospects for Development of Anti-Atherosclerosis Therapies

Due to the involvement of mitochondria in many aspects of atherosclerosis pathogenesis, they can be considered as a promising marker of the disease and direct therapeutic target for mitochondria-specific pharmacological agents (Table 2).

It is known that PGC-1α has a beneficial effect on the endothelial phenotype, however, it is not clear whether an increase in PGC-1α will be useful as a strategy against atherosclerosis. Epidemiological studies have shown that PGC-1α polymorphisms are associated with hypertension, carotid arteriosclerosis and coronary heart disease, suggesting the association of PGC-1α with vascular diseases. However, such observational studies do not prove the importance of reducing the mass or function of mitochondria in endothelium, given that PGC-1α regulates many other aspects of metabolism. In general, however, available studies suggest interventions that activate PGC-1α and stimulate mitochondrial biogenesis protect against CVD, and efforts are underway to identify small molecules that can be developed as drugs [27].

**Figure 3:**
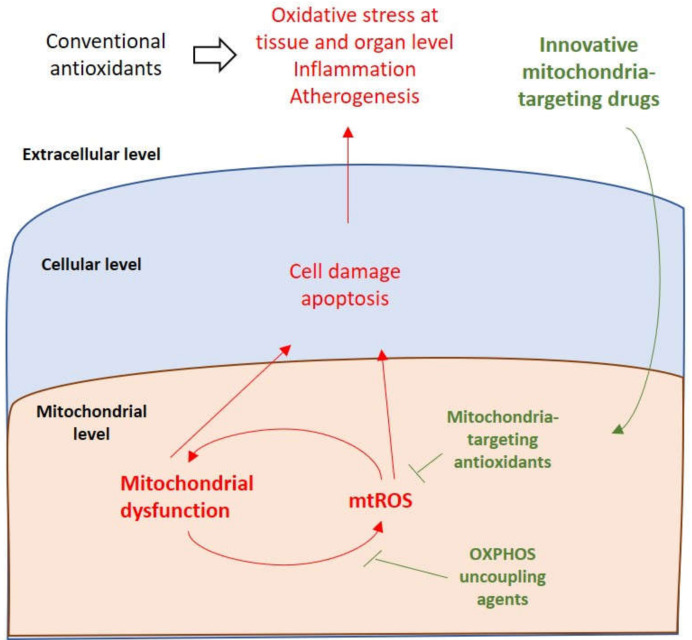
Mitochondria-targeting innovative therapies. While conventional antioxidants act at the organism level and often lack efficacy, mitochondria-targeting agents penetrate the cell to accumulate in the mitochondria, where they can neutralize (T-bars) mitochondrial reactive oxygen species (mtROS) at the very beginning of the pathogenic cascade or block their production breaking the vicious circle of mitochondrial dysfunction (arrows).

Antioxidants targeting mitochondria represent a new strategy for preserving vascular function and preventing cardiovascular disease. Weakened endothelium-dependent dilatation usually occurs due to a decrease in bioavailability of NO due to oxidative stress. Reducing mitochondrial oxidative stress is a potential opportunity to restore organelle and whole cell homeostasis and their functioning. The superior efficacy of mitochondrial antioxidants is explained by their preferential accumulation in the organelles that are major ROS generators (Figure 3). A promising mitochondria-targeted antioxidant is ubiquinone MitoQ, a modified form of the natural ubiquinone antioxidant combined with the lipophilic triphenylphosphonium cation, which allows the complex to penetrate through the mitochondrial membrane. A study conducted in 2014 showed the effectiveness of MitoQ in reducing vascular endothelial dysfunction in old mice. This improvement was associated with normalization of mitochondrial stress and mitochondrial health markers, such as PGC-1α, MnSOD and COX IV [73,74].

A recent review has presented a list of promising antioxidant agents in the treatment of atherosclerosis [75]. One of such antioxidants is resveratrol, a polyphenolic compound found in many edible plants. Resveratrol treatment has been shown to suppress palmitic acid damage in human umbilical vein endothelial cells (HUVECs), including cell viability damage, oxidative stress, and loss of mitochondrial membrane potential. In addition, resveratrol increases the level of mitochondrial fusion proteins (MFN1, MFN2 and OPA1) and also inhibits palmitic acid-induced mitochondrial fragmentation [56]. Quercetin is a flavonoid compound that has shown anti-inflammatory, antioxidant activity and affect lipid metabolism. Melatonin is also an antioxidant that eliminates ROS by stimulating mitophagy in macrophages. Curcumin is a pigment contained in turmeric that exhibits various biological activities, including antioxidant properties. This agent is present in in popular spices of Asian kitchen, and is traditionally regarded as a biologically active food component with beneficial effects for prevention and treatment of various conditions, especially those associated with inflammation. It should be noted, however, that dietary curcumin has relatively low bioavailability, so preparations with improved properties are needed for attaining better effects [76]. Curcumin has been shown to reduce the synthesis of pro-atherogenic cytokines, including interleukin (IL)-1, TNF-a in human monocytes and induces anti-inflammatory M2 polarization of mouse macrophages [75].

A recent study has demonstrated antioxidant properties of luteolin, a flavonoid known for its positive effect on the cardiovascular system, in HUVECs. The study reported that pre-treatment with luteolin significantly reversed oxidative stress signs in a dose-dependent manner. In addition, collapse of the mitochondrial membrane potential, p53 phosphorylation, decrease in the Bcl-2/Bax ratio in the mitochondrial membrane, and release of cytochrome c from the mitochondria, were all suppressed by luteolin treatment [77].

Dietary components represent only one of the many options for ameliorating mitochondrial oxidative stress. There is strong evidence that controlling known risk factors of CVD can help alleviate the mitochondrial stress. Smoking cessation is one of the prominent examples. Furthermore, correction of obesity and proper control of diabetes mellitus should certainly be listed as potent measures to prevent mitochondrial stress and associated aggravation of CVD [78].

The development of mitochondria-targeting therapies is currently ongoing. For a chronic condition such as atherosclerosis, it is important to develop interventions with long-term efficacy. Therefore, gene therapy and nanotechnology are considered as new promising approaches [79]. Modification of drug delivery method to improve its availability in the mitochondria is another line of research. For instance, curcumin is a hydrophobic substance with limited bioavailability, that needs a special delivery system. It has been shown that nanosuspension provides a high bioavailability of curcumin, and in vivo experiments have shown amelioration of the symptoms of atherosclerosis using curcumin nanosuspensions sonodynamic therapy [79]. Despite all the difficulties of using antioxidant therapy, some drugs are already involved in clinical trials. Therefore, MitoQ has shown its effectiveness in stages 1 and 2 of clinical trials with oral administration at a dose of 1 mg/kg, which makes it a promising drug for the treatment of diseases associated with mitochondrial dysfunction [80].

Thus, a new approach to the use of antioxidant therapy, which has proved its effectiveness, is the competent combination of antioxidants with excipients and the use of special technologies.

## 10. Conclusions

There is currently no doubt that mitochondrial dysfunction and mtDNA mutations play an important role in the pathogenesis of atherosclerosis Accumulating evidence demonstrates the involvement of dysfunctional mitochondria and mtDNA in the main aspects of atherogenesis: vascular inflammation, cell death, aggregation of oxLDL, defective mitophagy, and oxidative stress. In addition, possible precursors of atherosclerosis, for example, hypercholesterolemia and the aging process, are associated with mitochondrial dysfunction. Currently, ROS are considered as the main factor in damage to mitochondria and mtDNA. However, data are accumulating indicating the appearance of defective mtDNA at an early stage of atherosclerosis, when the oxidative status of cells is not yet prominent. These data bring us closer to the necessity of studying other regulators (initiators) of mtDNA damage to expand the possibilities of treatment direction.

A promising therapeutic area is mitochondria-targeted antioxidant therapy. However, a number of difficulties hinder the rapid development, including limited bioavailability of drugs and need for fine-tuning of dosage, and method of delivery. Thus, continuing the search for sources of mtDNA damage could make a significant contribution to our understanding of the pathogenesis of atherosclerosis and affect the progress of its treatment.

## Figures and Tables

**Table 1:** mtDNA variants associated with atherosclerosis.

Source	mtDNA Variants			Reference(s)
Leukocytes and arterial wall tissue (post-mortem samples) from patients with atherosclerosis	m.204T>C	m.3243A>G	m.14459G>A	[63]
	m.228G>A	m.12315G>A	m.8251G>A	
	m.16223C>T	m.3336T>C	m.9477G>A	
	m.1719G>A	m.5178C>A	m.ins8528A	
	m.3010G>A	m.12705C>T	m.14709G>A	
	m.3256C>T	m.13513G>A	m15059G>A	
Peripheral blood cells from CAD patients	m.5725T>G	m.5568A>G		[64]
	m.12308A>G	m.5711A>G		
Various sources	m.617G>A	m.8794T>C	m.3316G>A	[65]
	m.3243A>G	m.8839G>C		

**Table 2:** Pharmacological agents targeting oxidative stress associated with mitochondrial dysfunction.

Name	Description	Effect	Reference(s)
PGC-1α	Peroxisome proliferator-activated receptor gamma coactivator 1-alpha - transcriptional coactivator	Favorable effect on the endothelial phenotype, stimulation of mitochondrial biogenesis	[27]
MitoQ	Modified mitochondria-targeted ubiquinone	Decreased vascular dysfunction, normalization of mitochondrial stress	[73,74]
Resveratrol	Antioxidant polyphenolic nature	Gene regulation, normalization of mitochondrial membrane potential, increased level of mitochondrial fusion proteins, inhibition of mitochondrial pharagmentation	[56,75]
Quercetin	Flavonoid antioxidant	Anti-inflammatory effect, effect on lipid metabolism, delayed development of atherosclerosis	[75]
Melatonin	Antioxidant	Disposal of ROS by activation of mitophagy in macrophages	[75]
Curcumin	Antioxidant polyphenolic nature	Decreased synthesis of proatherogenic cytokines, induction of anti-inflammatory polarization of macrophages	[75]
Luteolin	Flavonoid antioxidant	Protection against H_2_O_2_-induced oxidative stress, suppression of intracellular Ca^2+^ growth and cytochrome c release, normalization of mitochondrial membrane potential	[76]
